# Short-Term Attractive Tilt Aftereffects Predicted by a Recurrent Network Model of Primary Visual Cortex

**DOI:** 10.3389/fnsys.2019.00067

**Published:** 2019-11-08

**Authors:** Maria del Mar Quiroga, Adam P. Morris, Bart Krekelberg

**Affiliations:** ^1^Center for Molecular and Behavioral Neuroscience, Rutgers University, Newark, NJ, United States; ^2^Behavioral and Neural Sciences Graduate Program, Rutgers University, Newark, NJ, United States; ^3^Neuroscience Program, Department of Physiology, Biomedicine Discovery Institute, Monash University, Clayton, VIC, Australia

**Keywords:** V1, orientation, tilt aftereffect, adaptation, perception, recurrent connections, model

## Abstract

Adaptation is a multi-faceted phenomenon that is of interest in terms of both its function and its potential to reveal underlying neural processing. Many behavioral studies have shown that after exposure to an oriented adapter the perceived orientation of a subsequent test is repulsed away from the orientation of the adapter. This is the well-known Tilt Aftereffect (TAE). Recently, we showed that the dynamics of recurrently connected networks may contribute substantially to the neural changes induced by adaptation, especially on short time scales. Here we extended the network model and made the novel behavioral prediction that the TAE should be attractive, not repulsive, on a time scale of a few 100 ms. Our experiments, using a novel adaptation protocol that specifically targeted adaptation on a short time scale, confirmed this prediction. These results support our hypothesis that recurrent network dynamics may contribute to short-term adaptation. More broadly, they show that understanding the neural processing of visual inputs that change on the time scale of a typical fixation requires a detailed analysis of not only the intrinsic properties of neurons, but also the slow and complex dynamics that emerge from their recurrent connectivity. We argue that this is but one example of how even simple recurrent networks can underlie surprisingly complex information processing, and are involved in rudimentary forms of memory, spatio-temporal integration, and signal amplification.

## Introduction

In a broad sense, sensory adaptation is the phenomenon that perception depends not only on the current stimulus, but also what was presented before. Adaptation is found across a wide range of time scales, from contrast adaptation occurring within a few hundreds of milliseconds (Shapley and Victor, 1978; Heinrich and Bach, 2001) to slow serial dependence spanning beyond seconds (Chopin and Mamassian, 2012; Fischer and Whitney, 2014). These behavioral phenomena are of interest in terms of their function, but also as a tool to gain insight into the underlying neural mechanisms. Here, we focus on visual adaptation on the timescale of a few 100 ms. This has high ecological relevance as it corresponds to the typical duration of a single fixation in the primate.

It is well-known that exposure to an oriented stimulus (the “adapter”) affects the perceived orientation of a subsequent stimulus (the “test”). In behavioral experiments with such an adaptation protocol, subjects typically report that the test orientation is more different from the adapter than it really is. This is called the tilt aftereffect (TAE); the test is repulsed away from the adapter (Gibson, 1933). Functionally, this phenomenon is thought to reflect the visual system's constant adjustment and recalibration to improve its ability to discriminate visual inputs (Clifford et al., 2000; Krekelberg et al., 2006a; Kohn, 2007; Schwartz et al., 2007; Kristjansson, 2011).

In primary visual cortex, similar adaptation protocols result in two well-documented changes (Gilbert and Wiesel, 1990; Müller et al., 1999; Dragoi et al., 2000; Felsen et al., 2002; Wissig and Kohn, 2012; Patterson et al., 2013, 2014). First, neurons shift their preferred orientation. The dependence of these shifts on the properties of the adapter are complex, but repulsive shifts (away from the adapter) dominate at short time scales (Wissig and Kohn, 2012; Patterson et al., 2013, 2014). Second, neurons change their peak response to the test stimulus. Here too the neural changes are complex, but rate suppression is found when the adapter is small compared to the receptive field (Wissig and Kohn, 2012; Patterson et al., 2013, 2014).

Although tuning shifts and rate suppression are sometimes both described as being the consequence of plasticity (Yao and Dan, 2001; Felsen et al., 2002), we recently showed that tuning shifts could arise from the attractor dynamics imposed by a network's recurrent connections. We studied the dynamics of orientation-tuned units in a recurrently connected network model without any form of plasticity (i.e., no changes in the biophysical, intrinsic properties of neurons or their synaptic connections) and showed that this model quantitatively captured the tuning shifts observed in monkey and cat V1 (Quiroga et al., 2016). This model, however, could not account for rate suppression, which does appear to require a mechanism involving some form of plasticity (Sanchez-Vives et al., 2000a,b).

Several modeling studies have linked the neural to the behavioral phenomena. As has been noted previously (Gilbert and Wiesel, 1990; Yao and Dan, 2001; Teich and Qian, 2003; Jin et al., 2005), tuning curve repulsion predicts (perhaps counterintuitively) an *attraction* of the percept, contrary to the typical TAE. However, rate suppression predicts a *repulsion* of the percept. Hence, to account for the fact that the behavioral TAE is typically repulsive, we have to assume that, in a typical TAE experiment, rate suppression is more potent than tuning shifts (Jin et al., 2005; Ursino et al., 2008). In the current contribution, we use this link between neural and behavioral findings to generate behavioral predictions based on our model, and test them in healthy human subjects.

Specifically, we reasoned that a hypothetical V1 with recurrent connections but without plasticity (i.e., with tuning shifts, but without rate suppression) should lead to an attractive TAE, while a V1 with plasticity but without recurrent connections should lead to a repulsive TAE. Given that no experimental methods can block all plasticity or remove all recurrent connectivity, testing this prediction will necessarily be somewhat indirect. Our experimental test relies on two observations. First, our previous modeling results show that the influence of attractor dynamics plays a significant role for adaptation on a time scale of at most a few 100 ms. Second, experimental data in macaque V1 show that rate-suppression is a comparatively slow process; its magnitude is small when adaptation is brief, and it increases substantially on a time scale of several hundreds of milliseconds (Patterson et al., 2013). This leads to the prediction that the attractive TAE (caused by the recurrent connectivity) should dominate at short time scales. To test this prediction, we designed a novel variant of the TAE adaptation protocol that minimizes long-term adaptation. Our experiments show that the TAE in human subjects is indeed attractive on a time scale of <200 ms. In the discussion we return to the question of how this informs our view of early visual processing, and the role of recurrent connections in particular.

## Methods

All experimental procedures were approved by the local Institutional Review Board, followed the Declaration of Helsinki, and the National Institutes of Health's guidelines for the ethical treatment of human subjects. All subjects provided written informed consent.

### Apparatus

Stimuli were presented on a Sony FD Trinitron (GDM-C520) CRT monitor using custom software (Neurostim, http://neurostim.sourceforge.net). The display measured 40° (width) × 30° (height) at a viewing distance of 57 cm. Eye-position was monitored using a 500 Hz video-based eye tracker (Eyelink II; SR Research, Mississauga, Canada) and the subject's head was stabilized using a bite bar.

### Short-Term Adaptation Paradigm

Subjects were required to maintain fixation within a 3° × 3° square at the center of the display (around the fixation point) for the duration of each trial (excluding the response epochs). Each trial started when the subject fixated; trials in which subjects failed to fixate appropriately were terminated immediately, discarded, and repeated at a random later time within the block.

The trial started 250 ms after fixation with the presentation of the adapter (an oriented Gabor) on the left or right (selected randomly) and a null adapter (See section Visual Stimuli) on the other side. The null adapter was included to match spatial frequency and contrast adaptation on both sides of fixation and to maintain relative symmetry in the display (to prevent shifts of attention to one side). Immediately after, two oriented Gabors were presented for a variable duration, one on either side of the fixation point. Subjects were instructed to report which of these Gabors was tilted more clockwise. The Gabor that appeared in the same location as the adapter is referred to as the “test” (right Gabor in the example trial shown in **Figure 2A**), and the Gabor that appeared in the location of the null-adapter is the “reference” (left Gabor in the example trial shown in **Figure 2A**). After the presentation of the test and reference stimuli, two masks were presented in the same spatial locations on either side of the screen for 500 ms. These masks were identical to the null-adapters; they served to minimize afterimages and limit the amount of temporal integration of the target stimuli. Subjects indicated which Gabor (left/right) appeared more clockwise by pressing one of two designated keys on the keyboard.

In a traditional adaptation paradigm, the same (or similar) adapter orientation is repeated on every trial. In such a paradigm, effects can accumulate across trials and therefore mix the influence of mechanisms that operate on a short and long time scale (Discussion). Here, we minimized the contribution of long-term effects by choosing a new random adapter orientation on each trial (orientation drawn from a flat distribution between 0 and 180°), thereby spreading long-term orientation-specific effects equally across orientation space over the session. Under these circumstances, orientation-specific behavioral consequences of adaptation can only be ascribed to a mechanism that operates on the timescales of single trials.

Subjectively, this paradigm is substantially more difficult to perform than a standard orientation discrimination paradigm. The reasons for this include the variation of the reference across trials (which requires subjects to compare the two test stimuli on each trial), the brief duration of the test, and the potent masking stimuli. For this reason, all subjects practiced the task (without adapters) for at least two and up to 5 h prior to completing experimental trials. Auditory feedback was provided during these practice runs and the data were only used to assess whether the subject consistently performed the task.

#### Invisible Adapter TAE Experiment

Eight naïve subjects (three males, five female) participated. All participants had normal or corrected-to-normal vision, were aged between 17 and 36, and right-handed.

In this experiment, we used adapters that were high enough in spatial frequency that their orientations could not be resolved by the subjects (“invisible” adapters; see “Isolating Short-term Attractive Aftereffects” in Results for the rationale). The spatial frequency of the adapter Gabors was fixed for the duration of the experiment but varied per subject, according to their individual ability to perceive orientation at high spatial frequencies (based on the screening experiment, below). To avoid the necessity of using spatial frequencies beyond the limits of our monitor (resolution: 1,280 × 960 at a refresh rate of 90 Hz), the stimuli were placed at relatively high eccentricity (centers at 12 degrees of visual angle (dva) to either side of the fixation dot).

Both experimental data (Felsen et al., 2002) and our model (Quiroga et al., 2016) show that the largest tuning curve shifts occur when the difference between the adapter and the test is ~20°. Hence, to maximize the expected attractive TAE, we jittered the offset between the reference and the adapter using a uniform probability distribution between 17° and 23° or between −17° and −23°, all in randomly interleaved trials. In the main analysis, we ignored the small variation around the ±20° mean and grouped the trials based only the sign of the offset between the reference and the adapter (clockwise condition: +20° or counterclockwise condition: −20°).

We used an adaptive procedure (Kontsevich and Tyler, 1999) to choose the orientation offset between the test and reference Gabors in each trial and estimate the point of subjective equality. The adapting stimulus was presented for 200 ms.

To test subjects' ability to perceive the orientation of the adapter, we randomly interleaved (10%) catch trials. In these trials, the test Gabor was replaced by a null-adapter, leaving the adapter as the only oriented element on that side of the screen.

Trials were presented in blocks of 64 trials for each test duration: [50, 100, 200] ms. Each 15-min run consisted of four blocks, and subjects typically completed two to three runs in a 1-h session. Subjects completed between 6000 and 9000 trials (mean 7000) across multiple days.

#### Screening Experiment

We mapped each participant's ability to discriminate the orientation of two Gabors at 12 dva eccentricity as a function of spatial frequency. The task was identical to the TAE experiment except that there were no adapters and no masks (i.e., only the reference and test were shown). The spatial frequencies for the reference and test were chosen from [3, 4, 5, 6, 7, 8] cycles per degree of visual angle (cpd) across trials (though always matched on each trial). Because our goal was to measure the discriminability of the orientation of the adapter and the reference in the main experiment, the orientation offset between the test and the reference in the screening experiment was ±20° plus a random jitter of ±3° (following the same procedure as the TAE experiment). As for the other experiments, the subjects' task was to identify the most clockwise stimulus (left or right). The test was presented for 200 ms, matching the longest test in the TAE experiment, and thus provided an upper bound to visibility for all test durations.

From their responses, we determined two spatial frequencies per subject. The first was the highest spatial frequency for which they performed the task at least 80% correct; this was used for the test stimulus in the invisible adapter TAE experiment. The second was the lowest spatial frequency that was higher than that of the test and for which the subject was at or close to chance performance. This was used as the spatial frequency of the adapter in the invisible adapter TAE experiment. This ensured that the subject was unable to judge the orientation of the adapter stimulus accurately, while keeping the spatial frequencies of the adapter and test as similar as possible (see section Results for rationale).

#### Visible Adapter TAE Experiment

In this control experiment, the centers of the stimuli were positioned 3 dva to either side of the fixation dot. The spatial frequency of the sinusoidal modulations underlying both Gabors and null-adapters was 2 cpd. The orientation of the test was offset from that of the reference by −12, −8, −4, 0, 4, 8, or 12°, which allowed us to compute a psychometric curve. The adapting stimulus was presented for 100 ms. All other procedures matched those in the invisible adapter TAE experiment. Three subjects participated in this experiment. One subject could not reliably perform this task at the shortest test duration (50 ms), even for the largest differences between test and reference. Hence, we excluded this condition from the analysis for this subject.

### Visual Stimuli

The adapter, test, and reference stimuli were oriented Gabors (sinusoidal luminance gratings modulated by a Gaussian contrast envelope to fade smoothly into the background). The standard deviation of the Gaussian envelope was 0.8 dva, the contrast was 75%, and the phase of each was randomized independently on each trial. We constructed the null-adapters by adding together eight Gabors whose orientations spanned 180° evenly with a random phase offset. These null-adapters were matched in spatial frequency to the adapting and testing Gabors, but contained little orientation specific energy (**Figure 2**). The contrast of the component gratings in the null-adapters was adjusted to produce an approximate perceptual match to the contrast of the single Gabor stimuli. The fixation stimulus was a small red square located in the center of the screen, which remained visible for the duration of the trial. The mean luminance of the equal energy white screen was set to 30 cd/m^2^.

### Data Analysis

The adapter orientation was randomized between 0° and 180° across trials (to avoid accumulation of adaptation across the session; see above), but the reference and test orientation were defined relative to the adapter on each trial. This allowed us to analyze the data in the standard way, by calculating psychometric functions for adapters that were either clockwise or counterclockwise (relative to the reference). To estimate the lapse rate, we fitted a logistic psychometric function to the data [using the *psignifit* toolbox (Wichmann and Hill, 2001b)] while pooling across clockwise and counterclockwise adapters, separately for each subject. Then, we fitted the responses in the trials with clockwise and counterclockwise adapters separately, fixing the lapse rates to the previously estimated values. We defined the shift in perceived orientation (i.e., the TAE) as the difference between the points of subjective equivalence (PSE) in these two curves. A positive difference signals an attractive shift; that is, the perceived orientation of the test was biased toward that of the adapter. Statistical comparison of the PSE differences in individual subjects was performed using Monte Carlo simulations based on the response data, as implemented in the *pfcmp* function for Matlab (Wichmann and Hill, 2001a).

At the group level, we used the increased power of parametric tests (Student *T*-test, and repeated measures analysis of variance) after confirming that the dependent measures (the TAE) did not deviate significantly from normality using the Lilliefors test.

### Model

#### Recurrent Network and Dynamics

We implemented an artificial network consisting of a bank of orientation-tuned V1 units, each receiving weakly tuned feedforward input (representing V1 neurons' judicious selection of LGN inputs), and recurrent excitatory and inhibitory input from its neighbors (Carandini and Ringach, 1997; Teich and Qian, 2003). All model parameters were rooted in empirically observed measurements and the units exhibited typical V1-like tuning curves and response dynamics to isolated oriented stimuli. We list the essential model specifications here, but for full details see Quiroga et al. (2016).

Each of the 256 model neurons was modeled as a single passive voltage compartment, whose membrane potential *V*^θ^ obeyed the differential equation:

(1) $$\begin{array}{r} \tau \frac{dV^{\theta }}{dt}+V^{\theta }=V_{\text{lgn}}^{\theta }+V_{\text{cortex}}^{\theta }, \end{array}$$

with τ the membrane time constant, $V_{\text{lgn}}^{\theta }$ the synaptic potential generated by the thalamic inputs to the model neuron and $V_{\text{cortex}}^{\theta }$ is the net synaptic input to the neuron from its cortical neighbors (Carandini and Ringach, 1997; Teich and Qian, 2003). For each neuron, the input from LGN was a function of stimulus orientation ω and contrast *c*:

(2) $$\begin{array}{r} V_{\text{lgn}}^{\theta }\left(\omega ,c\right)=cJ_{\text{lgn}}f\left(\omega |\theta ,\kappa_{\text{lgn}}\right), \end{array}$$

where *J*_lgn_ represents the strength of the input and *f*(ω|θ, κ_lgn_) is a von Mises function with period π, mean θ, and concentration κ_lgn_

(3) $$\begin{array}{r} f\left(x|\mu ,\kappa \right){=}^{e^{\kappa \cos\left(2\left(x-\mu \right)\right)}}{/}_{2\pi I_{0}\left(\kappa \right),} \end{array}$$

and *I*_*o*_(κ)is the modified Bessel function of order zero. The recurrent connection profiles were modeled as the difference of an excitatory and inhibitory von Mises distribution:

(4) $$\begin{array}{r} F^{\theta }\left(\phi \right)=J_{\text{cortex}}\left(f\left(\phi |\theta ,\kappa_{E}\right)-r_{IE}f\left(\phi |\theta ,\kappa_{I}\right)\right). \end{array}$$

In this recurrent connection profile *J*_cortex_ represents the strength of the cortical connections, and *r*_*IE*_ the ratio of the strength of inhibition to the strength of excitation. With this connection profile we can define the recurrent input as:

(5) $$\begin{array}{r} V_{\text{cortex}}^{\theta }\left(t\right)=\underset{\phi }{\sum }F^{\theta }\left(\phi \right)R^{\phi }\left(t\right) \end{array}$$

The instantaneous firing rate was calculated as a piecewise linear function of the membrane potential:

(6) $$\begin{array}{r} R^{\theta }\left(t\right)=\alpha \max\left(V^{\theta }\left(t\right),0\right), \end{array}$$

with α a gain factor (i.e., increase in firing rate in spikes per second for a 1 mV increase in the membrane potential above zero). The model and all simulations were implemented in MATLAB and solved numerically using *ode45*, an adaptive time step Runge-Kutta method. Model parameters were determined in a non-linear optimization procedure that resulted in a quantitative match with the tuning curve shifts observed for brief duration adaptation in anesthetized macaque V1 (i.e., Figure 5 in Patterson et al., 2013). For details of this procedure see Quiroga et al. (2016). The parameters were: τ = 8 ms; α = 3.88 Hz/mV; *J*_lgn_ = 11.04 mV/Hz; κ_lgn_ = 0.47; *J*_cortex_ = 2.84 mV/Hz; *r*_*IE*_ = 1.24; κ_*E*_ = 1.12; κ_*I*_ = 0.56.

#### Rate Suppression

The recurrent network model reported previously (Quiroga et al., 2016) was developed specifically to isolate the role of recurrent connections and show that they are sufficient to explain dynamic tuning curve shifts even in the absence of all plasticity. Here we extended the model to investigate the potential interaction of recurrent dynamics with the changes in intrinsic properties that are also known to occur in the brain. In visual cortex, exposure to a stimulus is known to result in the reduction of the response to subsequent stimuli (Patterson et al., 2013). We modeled this as a reduction in rate that is proportional (with gain β) to the neuron's mean firing rate in the adaptation period 〈*R*^θ^〉 and recovers on an exponential time course with time constant ρ after the offset of the adapter at *t* = *t*_0_. Formally:

(7) $$\begin{array}{r} {R_{\text{adapt}}}^{\theta }\left(t\right)=R^{\theta }\left(t\right)-\beta \langle R^{\theta }\rangle e^{-\frac{t-t_{0}}{\rho }} \end{array}$$

## Results

We first present model simulations based on macaque V1 responses that lead to the prediction of a short-term attractive TAE, and then the results of a set of psychophysical experiments that test and confirm the prediction in human observers.

### Model Simulations

**Figure 1:**
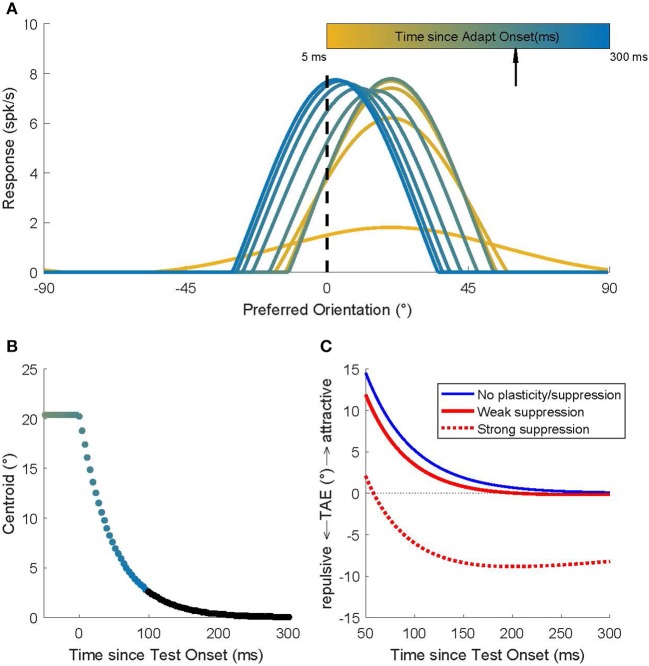
Recurrent network model simulations of an adaptation protocol in which a 20° adapter was followed by a 0° test. **(A)** Population activity dynamics in a model without plasticity. Color indicates progression over time. The earliest pattern (orange) represents the activity at the start (*t* = 5 ms) of the adaptation phase; the hill of activity is broad but, due to the recurrent connections, it gradually narrows around the neurons that prefer 20°. After 200 ms (black arrow) the 0° test stimulus is presented and the hill of activity gradually moves to position itself around the neurons that prefer 0°. **(B)** The dynamics of the location of the hill of population activity (here represented by its centroid). In the adaptation phase (*t* < 0 ms), the hill is centered on 20°. Once the 0° test is presented, the hill moves toward 0°, but this takes a surprisingly long time (Quiroga et al., 2016). Colors match those used to represent time in **(A)**. Time points omitted for clarity from **(A)** are shown here in black. **(C)** Predicted TAE in three models. One model (blue curve) has no plasticity and its tuning curve shifts are the consequence of the recurrent network dynamics alone. The other two models include the effect of spike rate suppression that occurs for prolonged (seconds) adapter presentations (red dashed curve; strong suppression) or for brief adapter presentations (red solid curve; weak suppression). These modeling results predict that the common long-term adaptation protocols should evoke the well-documented repulsive TAE, while an adaptation protocol that isolates (or emphasizes) the contribution of short-term effects should result in an attractive TAE.

We used a network model of orientation processing in V1 in which the neurons' weak orientation selectivity that arises from the afferent input is sharpened by strong, Mexican-hat type, recurrent intracortical connectivity (see section Methods). Figure 1A shows the population activity dynamics in the model for a simulated experiment in which an adapter with orientation 20° is first presented for 200 ms and then followed immediately by a 0° test stimulus. At the start of the adaptation phase (yellow; *t* = 5 ms), the hill of activity is broad, reflecting the tuning that arises from the combination of LGN input. While the adapter is on the screen (*t* < 200 ms) the hill of activity grows and narrows under the influence of the recurrent connections.

When the 0° test stimulus is shown (*t* = 200 ms), the LGN input is switched immediately (afferent delays to the LGN are ignored in this simulation) to provide maximal input to the unit that prefers 0°; and yet, the hill of activity in V1 moves only gradually toward the neuron that prefers 0°. Quiroga et al. (2016) showed that these population dynamics can be surprisingly slow, even though all neurons in the network have short membrane time constants (here 8 ms; see section Methods). The traveling speed of the hill of population activity is a complex function of the network connectivity, but using parameters estimated from macaque V1 (Patterson et al., 2013), it takes several hundreds of milliseconds after test stimulus onset before the hill reaches its stable state (Figure 1B). For a full discussion of the model, and how it captures the magnitude and dynamics of tuning curve shifts observed in cat and monkey V1, we refer to Quiroga et al. (2016). Here we focused on behavioral predictions of the model.

We used a labeled line readout to translate population activity into a (predicted) perceived orientation. Specifically, the decoded orientation was defined as the sum of the preferred orientations of the neurons, weighted by their instantaneous firing rate. To match the experimental protocol (see below), we did this separately for a 20° adapter and a −20° adapter and defined the TAE as the difference in the model's perceptual readout for these two conditions (Figure 1C; blue curve).

By design, the model of Quiroga et al. (2016) captured only the influence of network dynamics and not the changes in intrinsic neuron properties that can lead to a suppression of firing after adaptation. Hence, in a model without plasticity (blue curve in Figure 1C), one would predict an attractive TAE with a magnitude <15 degrees (the value early after test stimulus onset), and because the curve almost reaches zero in 300 ms, the duration of the attractive TAE should be <300 ms.

**Figure 2:**
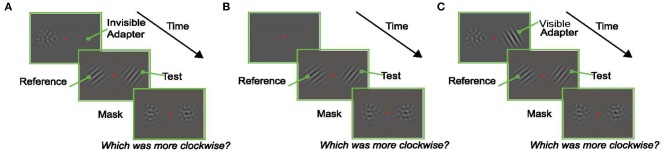
Experimental Paradigms. In each experiment, subjects fixated a central red dot for the duration of the trial. At the end of the trial they reported which of the oriented Gabors in the test display (left or right) was tilted more clockwise. **(A)** An example trial in the Invisible Adapter TAE experiment. An oriented pattern and a non-oriented mask appeared on either side of the fixation stimulus. The adapter's orientation was chosen at random in each trial to avoid the build-up of plasticity effects across trials, and the spatial frequency of the adapter was chosen such that the subject could not reliably perceive the orientation of the adapter (i.e., it was “invisible”). Immediately after the adaptation phase, an oriented test stimulus appeared on the same side as the adapter while a reference stimulus appeared on the opposite side. Finally, non-oriented masks were presented in both locations. **(B)** An example trial from the screening experiment. A reference and test stimulus with matching spatial frequencies, but an orientation difference of 20°, were shown for 200 ms. **(C)** Control experiment which followed the design shown in **(A)** with the exception that the adapter now had the same low spatial frequency as the test stimulus and was therefore visible to the subject.

However, rate suppression clearly does occur during adaptation; we extended the model to quantify the contribution of rate suppression. We made no specific assumptions about the underlying cellular mechanisms, but assumed that each neuron's firing rate suppression was proportional (β) to its average response to the adapter (i.e., we assumed that suppression was tuned) and that this response suppression recovered with an exponential (ρ) time course (Methods). We estimated these parameters based on Figures 2, 5 in Patterson et al. (2013). For adaptation periods on the order of seconds, the Patterson et al. data show that rate suppression is substantial (β = 50%) and recovery slow (σ = 500 ms). Entering these parameter estimates into the model leads to the prediction of a repulsive TAE (“strong suppression;” Figure 1C; red dashed line). This is consistent with behavioral findings and conceptually similar to the explanation of the repulsive TAE by Jin et al. (2005) and Teich and Qian (2003). For adapters that are presented for at most a few 100 ms, however, rate suppression in V1 is small (β = 20%) and recovers rapidly (ρ = 100 ms). Therefore, the model predicts an attractive TAE (“weak suppression;” Figure 1C; red solid line), for adaptation protocols that isolate short-term adaptation.

### Isolating Short-Term Attractive Aftereffects

To measure a short-term attractive TAE, we need to address two important issues. First, to emphasize short-term effects, the design should minimize the accumulation of adaptation both within a trial but also across trials. We addressed this by using brief adapters, and, unlike common TAE protocols, a random adapter orientation on each trial.

More specifically, subjects were instructed to respond which of two oriented Gabors (presented on each side of the fixation stimulus) was tilted more clockwise. One of these Gabors (the “test”) was preceded by an oriented adapter at the same spatial location, whereas the other (“reference”) was preceded by a non-oriented stimulus (null-adapter; see section Methods). Importantly, the orientation of the adapter was chosen randomly on each trial (i.e., any angle between 0 and 180°). The reference was on average oriented either 20° clockwise or 20° counterclockwise to the adapter orientation; this offset generates the largest tuning curve shifts in V1 and in the model (see section Methods). Randomization of the adapter orientation minimized the build-up of plasticity effects (i.e., rate suppression) across trials. This paradigm therefore allowed our measurements to isolate perceptual effects that are induced on the time scale of adapter presentation (200 ms in this experiment, matching the simulations in Figure 1). We measured the recovery time scale of these effects by presenting the “test” stimulus for one of three durations: 50, 100, and 200 ms, which the model predicts to be associated with progressively weaker attractive aftereffects (Figure 1B).

The second issue addressed by our design was a potential substitution confound. Given the brief test stimulus presentations, it is conceivable that on some trials subjects could compare the adapter (rather than the test) to the reference orientation. From this, we would infer the presence of an attractive aftereffect, independent of any true influence of adaptation. To address this, we exploited the invisible adapter paradigm of He and MacLeod (2001). They used laser interferometry to create a grating stimulus directly on the fovea with a spatial frequency so high that its orientation was invisible to the participants. Such invisible patterns nevertheless evoked a TAE in test stimuli with a lower spatial frequency (and therefore visible orientation) (He and MacLeod, 2001; Rajimehr, 2004). This offers an opportunity to study the TAE without the presence of the substitution confound. Our experiment was analogous to He and MacLeod, except that, lacking an interferometer, we presented the gratings at high eccentricity where even the orientation of gratings with more modest spatial frequencies cannot be resolved. Although strictly speaking only the orientation of these patterns was invisible, we refer to these patterns as invisible adapters.

### Screening Experiment

**Figure 3:**
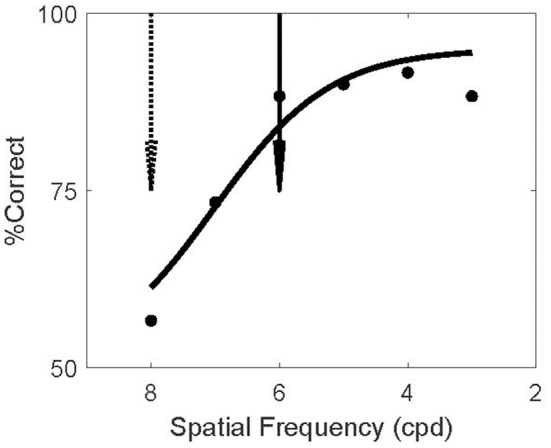
Screening Invisible Adapters. Psychometric curve for a single subject's performance on the screening task. This subject performed near chance for 8 cpd (dashed arrow) and above 80% correct for spatial frequencies above 6 cpd (solid arrow). Solid curve shows a logistic fit to the psychometric data. Based on this screening experiment, this subject's adapter frequency was set to 8 cpd and the test frequency to 6 cpd. Using the notation (8/6) to denote this outcome, two other subjects' screening experiments resulted in the same frequency setting outcome (8/6), four subjects had (7/4), and one subject had (6/4).

The screening experiment was designed to find such invisible patterns, separately for each subject. For each subject we assessed the ability to discriminate orientation as a function of spatial frequency. This experiment matched the test phase of the main experiments but no adapters or masks were present (because the adapters in the main study were not forward masked; Figure 2B). The example subject in Figure 3 could not reliably discriminate the orientation of 8 cpd patterns (dashed arrow). In the TAE experiment of this subject, the spatial frequency of the adapter was therefore set to 8 cpd; thus minimizing the possibility that the subject could report the orientation of the adapter.

In the model, the TAE results from the recurrent connections between neurons representing the adapter and the test. In V1 such connections are likely stronger between neurons with similar spatial frequency preference (Ts'o et al., 1986; Malach et al., 1993). Hence the model predicts a larger TAE if the test stimulus has a high spatial frequency similar to the adapter, while the logic of the experiment requires that the test stimulus spatial frequency is low enough to make it visible. We compromised between these conflicting demands by setting the test frequency to the highest spatial frequency at which the subject could reliably perform the task in the screening experiment (>80% correct). For the example subject this was 6 cpd (solid arrow).

### Invisible Adapter TAE Experiment

**Figure 4:**
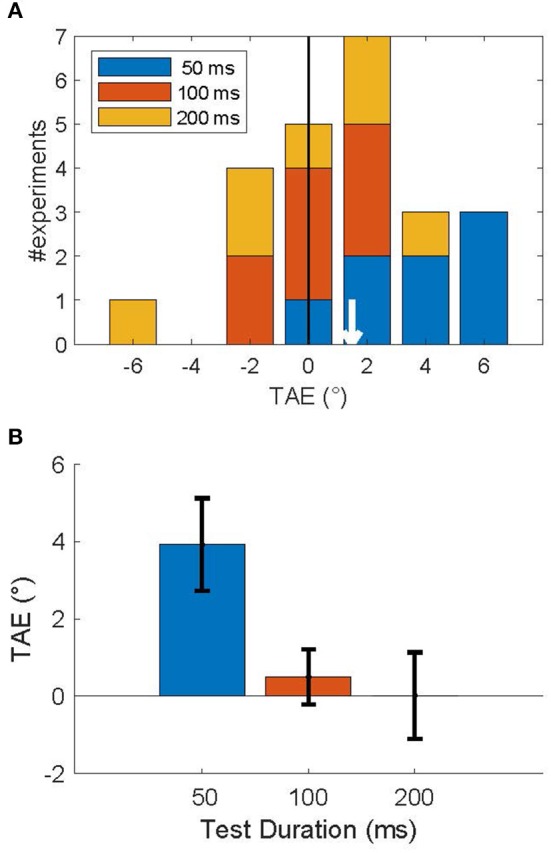
Attractive tilt aftereffects following an invisible adapter. **(A)** Histogram of TAE magnitude, across subjects for each test duration (legend). The mean TAE (white arrow) was attractive: 1.48°. **(B)** Average TAE per test duration. Error bars show the standard error in the mean. This figure shows that an adapter presented for 200 ms induced an attractive TAE that lasted <200 ms.

Figure 4A shows a stacked histogram of TAE sizes for all subjects. The overall mean TAE was 1.5°, which was significantly larger than zero (*p* < 0.05; one-sided *T*-test), and confirms our prediction that the TAE for brief (200 ms) adapters is attractive for a short time after (≤200 ms). This overall measure, however, averages over an underlying decay time-course that is seen more clearly in the average across subjects in Figure 4B. Statistical analysis confirmed a significant effect of duration on the TAE [*F*_(2)_ = 38, *p* < 0.001; one-way repeated measures ANOVA], reflecting the rapid recovery of the TAE on a 200 ms time scale. *Post-hoc* tests showed that only the shortest duration test (50 ms) led to a significant positive TAE (*p* < 0.01; one-sided *T*-test). For this shortest test duration, the effect was 3.9° in magnitude. TAE at test durations of 100 and 200 ms were not significantly larger than zero (*p* > 0.5), nor were they significantly different from each other (*p* > 0.7). At the single subject level, the TAE at the shortest duration was attractive for all but one subject, and significantly larger than zero in 4 out of 8 subjects (*p* < 0.05; Monte Carlo comparison of PSEs; see section Methods). No subjects had a significantly repulsive TAE.

**Figure 5:**
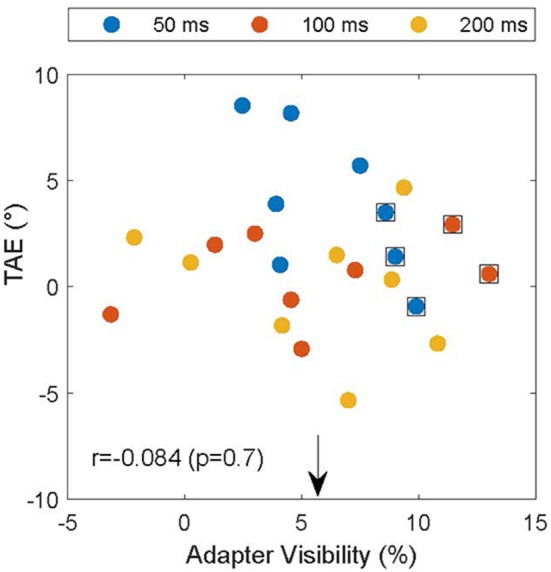
TAE as a function of the visibility of the adapter in catch trials. Individual TAEs are shown for all subjects and durations (legend) as a function of the visibility of the adapter on catch trials in matched conditions. The arrow indicates the mean visibility (5.7%), and data points surrounded by black squares indicate conditions in which the null hypothesis that the adapter was invisible could not be rejected (*p* < 0.05). TAE and adapter visibility were not significantly correlated (*r* = −0.084; *p* = 0.7), suggesting that the subjects indeed reported the orientation of the test and not, inadvertently, that of the adapter.

The experiment was designed to make the adapter's orientation invisible and thereby remove the potential confound that the subjects reported based on the orientation of the adapter instead of the test. However, due to drifts in subject thresholds, it is possible that subjects performed near chance for a high spatial frequency in the screening experiment, but were still able to detect the pattern's orientation in the TAE experiment. To assess whether this could account for the attractive TAE, we used interleaved catch trials in which the test grating was replaced by a null-adapter. In these trials the adapter was the only oriented element on the test side of the display. If the subjects could see the adapter, they would likely respond clockwise if the adapter was clockwise to the reference. We used this to estimate adapter visibility by calculating the proportion of trials in which the subject's report (CW vs. CCW) was consistent with the relation between the adapter and the reference. The extent to which these proportions differed from 50% provides a measure of adapter visibility. Figure 5 shows the relationship between the TAE and adapter visibility. On average, adapter visibility was 5.7 ± 4.2% (mean ± standard deviation), suggesting little awareness of adapter orientation, as intended by our design. For five of the data points (data points surrounded by black squares in Figure 5), we could not reject the null hypothesis that the adapter was invisible (*p* < 0.05, binomial test). These data points, however, did not drive the main results illustrated in Figure 4. Notably, even after excluding these data points, the mean TAE was significantly larger than zero (*p* < 0.05; one-sided *T*-test) and the TAE depended significantly on adapter duration [*F*_(2)_ = 23; *p* < 0.001]. Furthermore, if inadvertent adapter visibility caused the attractive TAE, it should be larger in conditions with high adapter visibility. Figure 5, however, shows that the magnitude of the TAE did not depend on the visibility of the adapter (Pearson correlation *r* = −0.084, *p* > 0.69).

### Visible Adapter TAE Experiment

**Figure 6:**
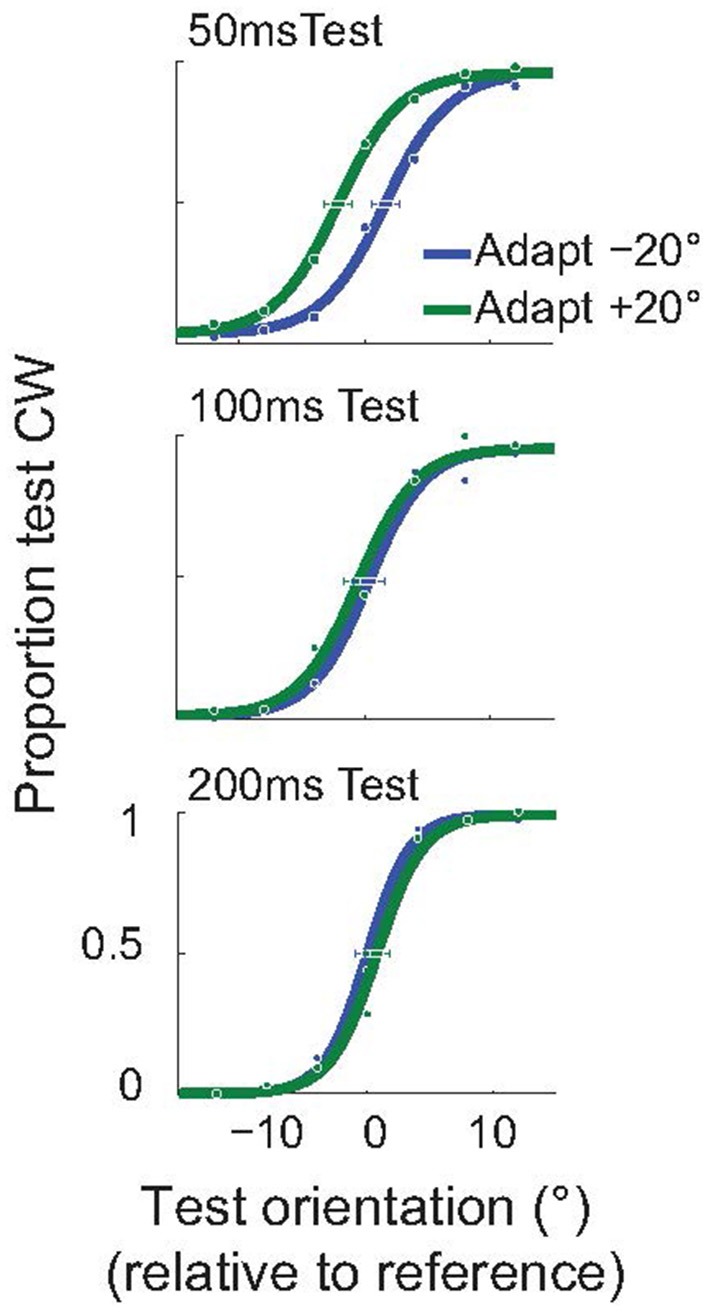
Short-term attractive tilt aftereffects with visible adapters. Psychometric curves for one example subject, for three test durations: 50, 100, and 200 ms. For the shortest duration of the test stimulus (50 ms), the perceived test orientation was significantly attracted toward the adapter. Horizontal error bars show 95% confidence intervals on the estimated PSE.

As explained above, we used an invisible adapter to avoid the possibility that the subjects reported the orientation of the adapter, instead of the test. For completeness, and to demonstrate that the attractive TAE is not restricted to the use of an invisible adapter, we performed a control experiment using a standard, visible, adapter. In this experiment all Gabor stimuli (adapter, test, null) had a spatial frequency of 2 cpd, and were presented at 3° eccentricity. Subjects performed the same orientation discrimination task (Figure 2C), but we used the method of constant stimuli to obtain better estimates of the full psychometric functions. In this experiment, the adapter was presented for only 100 ms (compared to the 200 ms used in the invisible adapter experiment). The example subject whose results are shown in Figure 6 had a significant, attractive TAE for a 50 ms test duration (*p* < 0.05; Monte Carlo simulations; see section Methods), but not for the longer test stimuli.

**Figure 7:**
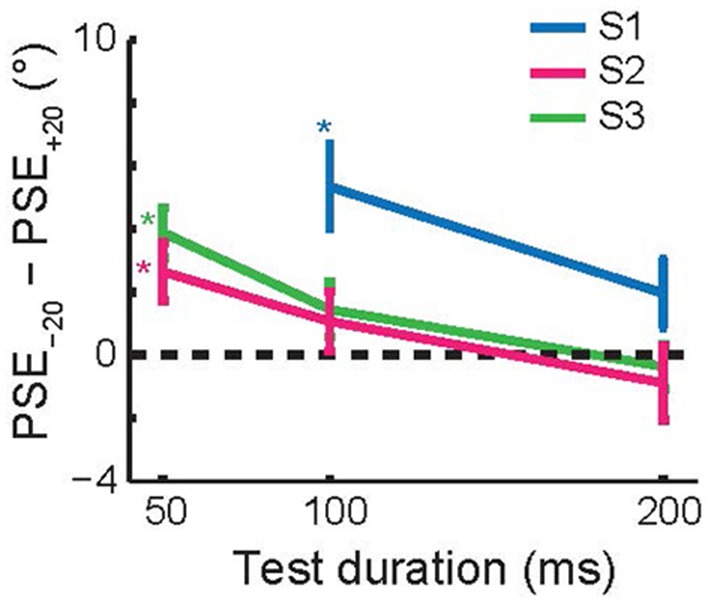
Recovery time course of short-term tilt aftereffects. The figure shows the change in PSE as a function of the test duration. For each of the subjects, the aftereffect was attractive at the shortest test duration at which they reliably performed the task (50 or 100 ms). Error bars show 95% confidence intervals based on Monte Carlo simulations (see section Methods).

Figure 7 shows the average size of the TAE across the three subjects that performed this experiment, as a function of test duration. Each subject had a significant, attractive TAE at the shortest test duration for which they could perform the task (*p* < 0.05, Monte Carlo simulations; see section Methods). This result is consistent with the findings for invisible adapters (Figure 4); brief adaptation results in an attractive TAE that decays on a time scale faster than 200 ms.

As explained above, in this experiment, we cannot exclude the possibility that subjects inadvertently reported the orientation of the adapter. We note, however, that the full psychometric curves (e.g., Figure 6) suggest that subjects primarily reported the orientation of the test (as instructed) and not the adapter. Notably, if the subject always reported the adapter orientation, the psychometric curves would have been independent of the test orientation (i.e., the figure would have shown two flat lines with the green line (a clockwise adapter) above the blue line (counterclockwise adapter). The sigmoidal shape of the subjects' psychometric functions showed that they responded primarily to the test stimulus, not to the adapter. Given, however, that this potential confound limits the forcefulness with which these data can be interpreted, we did not pursue this experimental design with a larger number of subjects.

## Discussion

Our experiments show that briefly presented oriented stimuli are perceived to be more similar to immediately preceding stimuli than they really are. This effect is opposite to the well-known (repulsive) tilt aftereffect. Under the conditions of these experiments, it is induced on a time-scale of 100–200 ms and recovers on a similar time scale: it is a short-term attractive tilt aftereffect. The attractive nature of the aftereffect and its fast recovery were predicted by our recurrent network model and therefore supports the view that orientation tuning curve shifts in V1 on the order of hundreds of milliseconds may be generated by the attractor dynamics of recurrent networks.

### Attractive and Repulsive Tilt Aftereffects

Gibson and Radner (1937) showed that 1 min of exposure to an adapter leads to a sizable tilt aftereffect. Even in that first paper, both repulsive and attractive aftereffects were reported. Repulsion was found when the orientation difference between the adapter and the test was between 10°and 30° (the so-called direct TAE), while attraction was observed in trials with large orientation differences (50°-90°; the indirect TAE). Our model predicted an attractive TAE even for small orientation differences, hence we focused on the direct TAE where the prediction appeared to conflict with well-established findings of a repulsive TAE even for brief test stimuli (Sekuler and Littlejohn, 1974; Wolfe, 1984). The apparent conflict is resolved by distinguishing two, logically distinct time scales involved in adaptation.

The first is the time scale of recovery from adaptation. This time scale can be probed, for instance, by leaving a gap between adapter and test or by varying the duration of the *test* stimulus. Previous studies have probed this recovery timescale extensively and concluded that shorter test stimuli generate larger TAEs (Wolfe, 1984; Magnussen and Johnsen, 1986; Harris and Calvert, 1989; Wenderoth and van der Zwan, 1989). The horizontal axes in Figures 4B, 7 represent this recovery time scale, and these figures show that our findings are consistent with those previous reports; short test flashes produce larger aftereffects.

The second is the time scale of the induction of adaptation. The duration of the adapter partially determines this time scale and previous work has shown that TAE magnitude increases with longer induction (Magnussen and Johnsen, 1986). Importantly, the duration of the adapter on a single trial is not the only determinant of the time scale of induction. In fact, Magnussen and Johnsen (1986) showed that multiple short presentations of the same adapter generate more adaptation than a single long presentation with the same total duration. This demonstrates that—as long as the adapter stays the same—adaptation accumulates over multiple trials (This is likely part of the reason why the commonly used top-up paradigm generates large adaptation effects even with brief top-up adapters). In previous work, the same adapter (or a small set of adapters) was presented repeatedly. This allowed such paradigms to tap into induction mechanisms operating on slow time scales, even when individual adapters were presented only briefly (Sekuler and Littlejohn, 1974).

In our experimental paradigm, adapter orientation was randomized across trials. Therefore, adaptation could not accumulate across trials in an orientation specific manner, and this isolated the TAE that is generated on a short induction time scale. There is therefore no contradiction between our finding of an attractive (direct) TAE and previous reports of a repulsive (direct) TAE; the separate induction time scales account for the difference.

### Time Scales

Although the time scale of the attractive TAE may seem short in terms of traditional behavioral adaptation experiments, it is actually well-matched to natural behavior. During normal exploratory behavior, primates make eye movements two to three times per second, hence new inputs arrive in the visual system on a time scale of hundreds of milliseconds (Ibbotson and Krekelberg, 2011). Our findings suggest that the V1 recurrent network is well-suited for the need to integrate and amplify information available within a typical fixation period.

Of course, recurrent network dynamics are unlikely to be responsible for neural and behavioral response changes on very long time scales. For instance, training on an orientation discrimination task results in tuning changes in early visual cortex (Schoups et al., 2001; Ghose et al., 2002). The link between these neural changes and behavioral improvements [i.e., perceptual learning (Watanabe and Sasaki, 2015)] is only partially understood (Ghose et al., 2002; Teich and Qian, 2003) and models that incorporate plastic changes (Teich and Qian, 2003; Chelaru and Dragoi, 2008; Ursino et al., 2008) are needed to understand this link. In a recurrent network, however, changes in connectivity can cause substantial changes in network dynamics (e.g., Figures 3, 4 in Quiroga et al., 2016). This shows that changes taking place on the slow time scale of learning can influence dynamics at the rapid sub-second time scale. Considering this aspect may help to generate testable predictions for changes in neural response properties and how these affect behavioral performance.

### Motion

Kanai and Verstraten (2005) reported repulsive motion aftereffects for long induction time scales, and attractive motion aftereffects for induction time scales below 200 ms. This phenomenological similarity of the short-term aftereffects in the motion and orientation domain is intriguing and suggests that similar recurrent network mechanisms could underlie these phenomena. This view is supported by theoretical and empirical work demonstrating that recurrent network dynamics could play a fundamental role in motion tuning (Mineiro and Zipser, 1998; Joukes et al., 2014; Pachitariu and Sahani, 2017).

Because there is substantial interaction between orientation and motion processing (Krekelberg et al., 2003; Kourtzi et al., 2008), one can also ask whether motion signals may lead to the short-term attractive TAE. In any adaptation paradigm, the successive presentation of the adapter and test has the potential to generate apparent motion signals, and theoretically, these could affect the perception of orientation. This would be analogous to the many ways in which translational motion can induce a misperception of position (Krekelberg, 2001; Krekelberg and Lappe, 2001; Müsseler et al., 2002). In our view, however, it is unlikely that the apparent motion signal played an important role in the perception of orientation in our experiments. First, the phase of the adapter and test were randomized independently, this should limit the strength of apparent motion. Second, in the invisible adapter experiment the spatial frequency of the adapter was higher than that of the test. This further reduces the strength of apparent motion and yet, the attractive TAE was of a similar magnitude. Of course, these arguments do not preclude the possibility that stronger motion signals could affect orientation adaptation. In fact, our recent findings show that one link between the neural mechanisms of complex form and motion processing is their reliance on recurrent connectivity (Joukes et al., 2017). This predicts that some of the adaptation effects resulting from recurrent connectivity could affect both form and motion perception.

### Neural Mechanisms

Previously, we showed that our model can account quantitatively for the repulsive tuning curve shifts observed in V1 (Quiroga et al., 2016). Here, we show that these repulsive tuning curve shifts predict an attractive TAE. Although counterintuitive, this apparent contradiction follows directly from the labeled line code (Gilbert and Wiesel, 1990; Yao and Dan, 2001; Teich and Qian, 2003; Jin et al., 2005). Consider a neuron that normally prefers 0°–in any labeled line decoder, spikes from this neuron are always interpreted as evidence in favor of 0°. Across a population of labeled line neurons, the percept is given by the location of the peak or center of the population activity. The statement that “adaptation causes a repulsive tuning curve shift” means, for example, that after adaptation at −10°, the 0° neuron responds most strongly to a 2° stimulus. That implies, however, that for a 2° stimulus, the population activity is centered on 0°. And this, in turn, means that the 2° stimulus is decoded as 0°. In other words, the 2° stimulus is attracted to the −10° adapter. The inversion from repulsion to attraction occurs because the former describes tuning curves (the response of a single neuron to different stimuli; a theoretical construct that exists only across trials), while the latter describes population activity (the response of many neurons to a single stimulus; this construct exists in each trial and underlies perception). For a more extensive discussion of these issues in the context of speed perception see Krekelberg et al. (2006b).

In our model, repulsive tuning curve shifts (and therefore the attractive TAE) results from the persistence of neural activity evoked by the adapter. This persistence is determined by the strength of the recurrent connections (as discussed in detail by Quiroga et al., 2016) and results in a predicted recovery time scale of the aftereffect on the order of a few 100 ms (Figure 1). Given that the model parameters were determined solely from the macaque V1 responses, the match between the model prediction and the behavioral results is surprisingly good and supports our claim that recurrent connections could underlie this behavioral effect.

Quantitatively, the model (Figure 1) predicts a larger TAE than we observed in our subjects. Part of this could be explained by well-known, but uncontrolled experimental factors. For instance, occasional reductions in attentional focus would reduce adaptation (Rezec et al., 2004) and fixational instability during the brief presentation of the adapter could also reduce adaptation. Our data provide support for the latter hypothesis; the interquartile range of fixational eye positions during the presentation of the adapter was negatively correlated with the TAE (Pearson *r* = −0.7; *p* = 0.03).

At the same time, because the model parameters were constrained only by data obtained in the anesthetized macaque, a mismatch between the model and human behavior should not be too surprising. The magnitude of tuning curve shifts increases when excitatory lateral connections are narrowly tuned, when inhibitory connections are broadly tuned, or when the overall strength of lateral connections is large compared to the afferent thalamic input (Quiroga et al., 2016). Most likely, these parameters are not the same across species, or even across individuals, or across retinal location within an individual. This implies that a range of TAE magnitudes is to be expected, just as the magnitude of tuning curve shifts also varies considerably across studies (Dragoi et al., 2000; Felsen et al., 2002; Patterson et al., 2013).

It would be interesting to find better methods to constrain model parameters. In principle, it should be possible to use behavioral responses to sequences of oriented gratings to infer the underlying functional connectivity in a network model. However, this problem is ill-constrained and adaptation is a poor replacement for an electrode (Krekelberg et al., 2006a; Sawamura et al., 2006; Hegdé, 2009; Solomon and Kohn, 2014; Kar and Krekelberg, 2016). Nevertheless, we believe that progress could be made by using carefully tailored paradigms, and models that are constrained not only by behavioral data, but also by the properties of rapid adaptation at the single neuron level (Benucci et al., 2009).

### Function

In our model, the short-term attractive TAE can be attributed to repulsive tuning curve shifts, which are caused by the pushing and pulling of population activity in a network with Mexican hat shaped recurrent connectivity (Quiroga et al., 2016). This connectivity pattern has many potential benefits. For instance, it sharpens orientation tuning and amplifies weak signals (Carandini and Ringach, 1997; Hahnloser et al., 2002; Teich and Qian, 2003), or it can implement a statistical prior assumption that changes in the sensory input are typically small (Deneve et al., 2001; Schwartz et al., 2007). The attractive TAE could therefore be interpreted as a side effect of such mechanisms that optimize orientation processing. The fact that these side-effects are not immediately obvious from the model, but require detailed simulations and exploration, attests to the fact that even simple recurrent networks can generate surprisingly complex responses. Phrased more positively, recurrent networks can underlie highly complex functionality. For instance, recurrent networks are able to implement rudimentary forms of memory that are needed in motion detection (Joukes et al., 2014), generate sensitivity for higher-order statistics (Joukes et al., 2017), or amplify weak stimuli in a state-dependent manner (Rutishauser and Douglas, 2009). These are elementary computations that are useful in perception and cognition and may be a reason why recurrent connections are ubiquitous across cortex.

### Alternative Models

The attractive TAE can be seen as an example of temporal integration; the response to the test is integrated with the response to the adapter and therefore the test looks like the adapter. This is an appealing, simple phenomenological description, but our goal is to understand the underlying neural mechanisms. In other words, one can postulate that a hill of persisting activity represents the adapter in a set of orientation-tuned neurons, and the interaction of this persisting activity with activity generated by the test could result in an attractive aftereffect. This description, however, does not answer the question of how such persistence is generated. Our model provides one answer to this question: we propose that recurrent connections lead to persistence and underlie temporal integration. In the model, the recurrent connections are necessary to explain an attractive TAE that lasts 50–100 ms (Figures 4, 7) because without them persistence is short, as activity dissipates on the time scale of the membrane time constant (8 ms).

There may well be other answers to the question of what generates (or contributes to) the persistence and temporal integration. For instance, prolonged exposure to visual input could affect the state or dynamics of slow channels and thereby result in persisting activity, or at least subthreshold depolarization, on a time scale of several 100 ms in individual neurons. Experimental data from cat visual cortex, however, show that prolonged exposure to visual input hyperpolarizes neurons by opening K-channels, and reduces their spiking activity (Sanchez-Vives et al., 2000a,b). The model shows that such effects shorten the recovery time course, and, when spike rate suppression is large enough, result in a repulsive, not an attractive TAE (Figure 1C).

Another possibility is that exposure to the adapter strengthens lateral connections between V1 neurons. This can generate repulsive tuning curve shifts (Felsen et al., 2002) and would therefore also lead to attractive aftereffects. To explain our findings, however, one would have to assume that a single 200 ms presentation of an adapter is sufficient to change the effective synaptic connectivity between neurons. We are not aware of experimental data that support the existence of such rapid plasticity in V1. Instead, plasticity is typically observed after the repeated exposure to the same (pairs of) stimuli (Yao and Dan, 2001). Just like the traditional TAE paradigm, this potentially taps into slow mechanisms that accumulate over seconds, even if the individual stimulus presentations are brief.

Of course, the neural mechanisms underlying the TAE need not be restricted to V1. The orientation-specificity of the TAE, however, demonstrates that the integrators must be orientation-tuned, which argues for at least a cortical locus. In addition, the finding that the TAE occurs even without conscious awareness of the adapter orientation (as in our invisible adapter experiment) suggests that its neural locus is relatively early (pre-attentive) in visual processing (Clifford, 2014). While it is certainly possible, ultimately even desirable, to construct a model that spans all levels of visual processing, it is difficult to constrain such a model with currently available experimental data.

In summary, in Quiroga et al. (2016) we proposed that recurrent network connections could underlie tuning curve shifts in V1 and supported this model with electrophysiological data. Here we used the recurrent network model, fully constrained by neural data, to generate novel predictions about orientation perception. Our experiments confirmed the predicted attractive TAE. Because the model uses only well-supported patterns of recurrent connectivity, it is a parsimonious, mechanistic explanation of repulsive shifts in short-term tuning curves and the short-term attractive TAE. We emphasize, however, that neither the electrophysiological nor the current behavioral data prove that the model is correct, or that recurrent connections are necessary to explain these phenomena. Such proofs of necessity are fundamentally beyond the purview of models. Instead, model value derives from the ability to generate conceptually novel understanding of neural processing and experimentally testable hypotheses.

## Data Availability Statement

The datasets generated for this study are available on request to the corresponding author.

## Ethics Statement

The studies involving human participants were reviewed and approved by The Institutional Review Board of Rutgers University. The patients/participants provided their written informed consent to participate in this study.

## Author Contributions

MQ, AM, and BK designed experiments and edited the manuscript. MQ performed the experiments. MQ and BK developed the model, performed model simulations, and analyzed the data. BK wrote the manuscript.

### Conflict of Interest

The authors declare that the research was conducted in the absence of any commercial or financial relationships that could be construed as a potential conflict of interest.
